# Soluble Toll-Like Receptor 4 Impairs the Interaction of Shiga Toxin 2a with Human Serum Amyloid P Component

**DOI:** 10.3390/toxins10090379

**Published:** 2018-09-18

**Authors:** Maurizio Brigotti, Valentina Arfilli, Domenica Carnicelli, Francesca Ricci, Pier Luigi Tazzari, Gianluigi Ardissino, Gaia Scavia, Stefano Morabito, Xiaohua He

**Affiliations:** 1 Department of Experimental, Diagnostic and Specialty Medicine, University of Bologna, Via San Giacomo 14, 40126 Bologna, Italy; arfilli.f@libero.it (V.A.); domenica.carnicelli@unibo.it (D.C.); 2 Immunohematology and Transfusion Center, S. Orsola-Malpighi Hospital, Via Massarenti 9, 40138 Bologna, Italy; francesca.ricci@aosp.bo.it (F.R.); pierluigi.tazzari@aosp.bo.it (P.L.T.); 3 Center for HUS Control, Prevention and Management, Fondazione IRCCS Ca’Granda Ospedale Maggiore Policlinico, Via Commenda 9, 20122 Milano, Italy; ardissino@italkid.org; 4 European Reference Laboratory for Escherichia coli, Istituto Superiore di Sanità, 00161 Rome, Italy; gaia.scavia@iss.it (G.S.); stefano.morabito@iss.it (S.M.); 5 Western Regional Research Center, U.S. Department of Agriculture, Agricultural Research Service, 800 Buchanan Street, Albany, CA 94710, USA

**Keywords:** hemolytic uremic syndrome, HuSAP, Shiga toxins, Toll-like receptor 4, decoy receptors

## Abstract

Shiga toxin 2a (Stx2a) is the main virulence factor produced by pathogenic *Escherichia coli* strains (Stx-producing *E. coli*, STEC) responsible for hemorrhagic colitis and the life-threatening sequela hemolytic uremic syndrome in children. The toxin released in the intestine by STEC targets the globotriaosylceramide receptor (Gb3Cer) present on the endothelial cells of the brain and the kidney after a transient blood phase during which Stx2a interacts with blood components, such as neutrophils, which, conversely, recognize Stx through Toll-like receptor 4 (TLR4). Among non-cellular blood constituents, human amyloid P component (HuSAP) is considered a negative modulating factor that specifically binds Stx2a and impairs its toxic action. Here, we show that the soluble extracellular domain of TLR4 inhibits the binding of Stx2a to neutrophils, assessed by indirect flow cytometric analysis. Moreover, by using human sensitive Gb3Cer-expressing cells (Raji cells) we found that the complex Stx2a/soluble TLR4 escaped from capture by HuSAP allowing the toxin to target and damage human cells, as assayed by measuring translation inhibition, the typical Stx-induced functional impairment. Thus, soluble TLR4 stood out as a positive modulating factor for Stx2a. In the paper, these findings have been discussed in the context of the pathogenesis of hemolytic uremic syndrome.

## 1. Introduction

Shiga toxins (Stx) are powerful exotoxins involved in the pathogenesis of severe illnesses in children, such as hemorrhagic colitis and hemolytic uremic syndrome (HUS), related to infections by pathogenic *Escherichia coli* strains (Stx-producing *E. coli*, STEC) [1,2,3]. These bacteria colonize the human gut after ingestion of contaminated food and intimately adhere to the epithelial lining of the intestine where they release Stx [1,2,3]. Upon absorption into circulation, Stx bind to several circulating cells (precocious toxaemia) [4,5] before targeting renal and cerebral endothelia which express the high-affinity receptor of the toxin, globotriaosylceramide (Gb3Cer) [6,7]. The latter event is the crucial point in the pathogenesis of HUS and occurs during advanced toxemia. Within cells, internalized Stx damage irreversibly ribosomes and DNA, causing the arrest of protein synthesis and the formation of apurinic sites in the nucleus [8,9]. As a consequence of the resulting endothelial injury/dysfunction, the formation of microthrombi occurs in renal glomeruli, causing the HUS triad, i.e. acute renal failure, mechanical damage of erythrocytes, and thrombocytopenia [1,3].

During precocious toxemia the multiple interactions with blood components would foment or prevent the onset of HUS. Gb3Cer is also expressed by monocytes and platelets which are targeted by Stx (reviewed in [4]). The binding of Stx to monocytes induces the release of pro-inflammatory molecules involved in HUS [5], whereas the interaction of Stx with platelets has been suggested to be directly related to thrombocytopenia [10]. Conversely, human neutrophils can recognize Stx by means of the pattern recognition receptor Toll-like receptor 4 (TLR4), which is also present on the membrane of monocytes and platelets [11]. In contrast to the latter cells, human neutrophils interact with Stx only via-TLR4 since they do not express the repertoire of enzymes necessary to synthesize the neutral glycolipid Gb3Cer [12]. The role of neutrophils is controversial since these cells have been considered protective (toxin sponge effect) or causal (toxin delivery to target cells) in the pathogenesis of HUS [4].

Among Stx, the subtype Stx2a is the most dangerous toxin produced by STEC which confers HUS [13], in spite of the presence of a specific inhibitor of this toxin in human blood, named human serum amyloid P component (HuSAP). HuSAP is associated to high density lipoproteins [14] and is present in soluble form at constant concentrations as a pair of pentagonally structured subunits [15,16,17,18]. Even low concentrations of HuSAP inhibit the toxic activity of Stx2a for target cells in vitro and protect mice from lethal effects induced by this toxin [15,18]. The interaction of HuSAP with Stx2a is mediated both by the A subunit and the B pentamer [16], whereas the A chain or the B-pentamer of the toxins are mainly responsible for the interaction with TLR4 on neutrophil membrane [19,20] or with Gb3Cer expressed by platelets, monocytes, and renal endothelial cells, respectively [4,21].

Upon stimulation of TLRs by pathogen-associated molecular patterns (PAMPs), the cells of the innate immunity are activated [22,23] and capable of releasing the decoy form of the corresponding TLR [24]. Soluble forms of TLR2 and TLR4 have been described in humans, which capture the specific stimulating PAMP hence reducing the responses of monocytes and neutrophils [25,26]. This mechanism is useful in preventing endotoxic shock by reducing the burden of pro-inflammatory cytokines released by monocyte/macrophages stimulated by the bacterial lipopolysaccharide via TLR4 (and the co-receptor MD2) [27,28,29]. The soluble decoy forms of TLR2 and TLR4 can be generated by (1) alternative mRNA splicing [30] or (2) conversion of the membrane receptor after endocytosis, followed by proteolysis in acidic compartments and subsequent exocytosis of the truncated form [25]. Recombinant soluble forms of the extracellular domain of TLR4 (70 kDa sTLR4) capable of dampening LPS-mediated TLR4-signaling have been obtained [28,29].

Since neutrophils and other innate immunity cells bind Stx through TLR4 and become activated after toxin stimulation, we have studied the effect of 70 kDa sTLR4 on the biological activity of these toxins and on the Stx2a/HuSAP interactions.

## 2. Results

### 2.1. Effect of the Soluble Recombinant Extracellular Domain of TLR4 on the Binding of Shiga Toxins to Neutrophils

**Figure 1:**
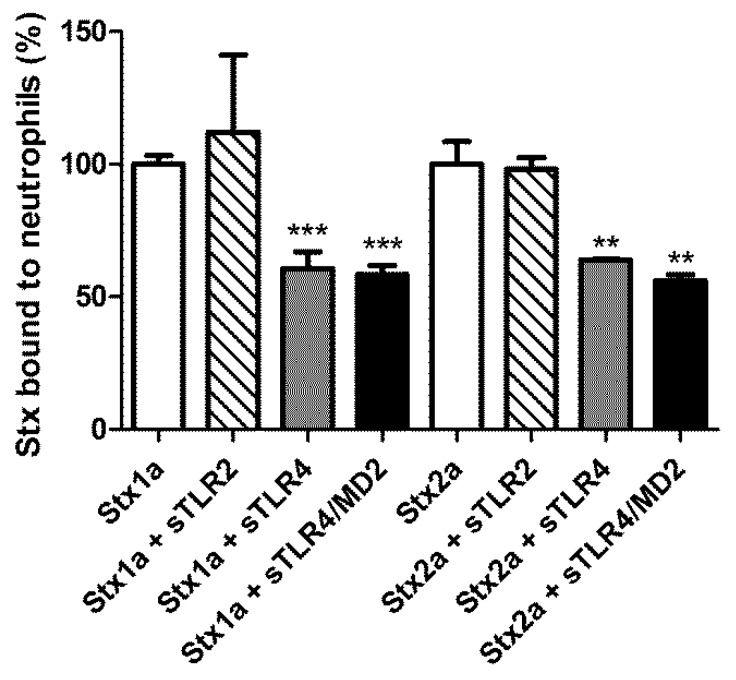
Inhibitory effect of the 70 kDa sTLR4 on the binding of Stx1a and Stx2a to human neutrophils. Neutrophils isolated from healthy donors were treated with Stx (60 nM) in the absence and in the presence of 600 nM of 70 kDa sTLR4, 70 kDa sTLR4/MD2, or sTLR2. The extent of binding of Stx to neutrophils was measured by indirect flow cytometric analysis as described in Materials and Methods (Section 4.3). The mean channel values of fluorescence (MCVs) of neutrophils treated with Stx1a or Stx2a were (mean ± SD, *n* = 3) 4.35 ± 0.14 or 3.90 ± 0.33, respectively. The data shown in the figure are expressed as binding percentages (mean ± SD, *n* = 3, unpaired *t*-test) *** *p* < 0.0001, ** *p* < 0.001.

Human neutrophils from healthy donors were treated with 60 nM Stx1a and Stx2a to reach full saturation of receptors [31]. The presence of 10-fold excess of 70 kDa sTLR4 inhibited the interaction of both toxins with neutrophils (Figure 1), further confirming the identity of TLR4 as a Stx receptor. The presence of the co-receptor MD2 did not significantly modify the inhibitory power of the 70 kDa sTLR4. The effect of 70 kDa sTLR4 on the binding of Stx to neutrophils is specific since the decoy form of TLR2 [24] was not able to confer the protection.

### 2.2. Effect of the Soluble Recombinant Extracellular Domain of TLR4 on the Activity of Shiga Toxin 2a on Target Cells

**Figure 2:**
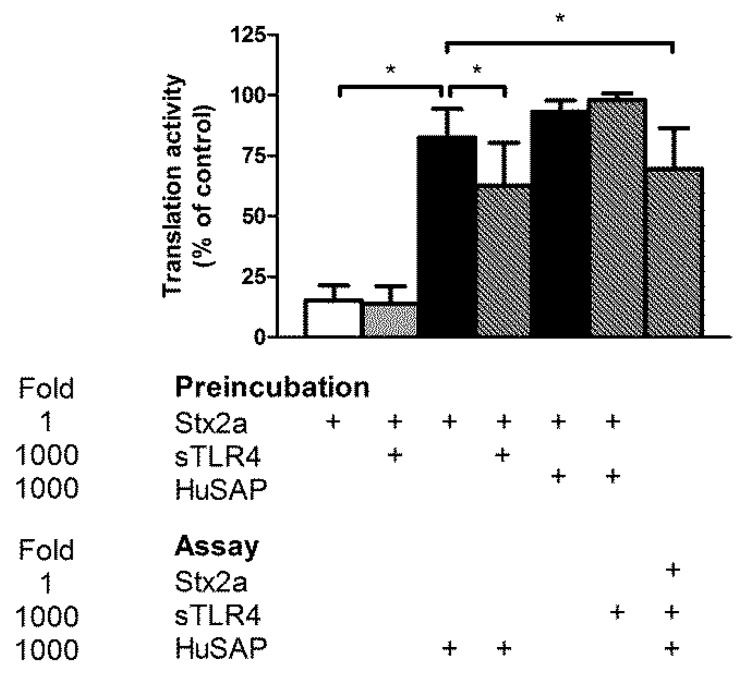
Effect of HuSAP and 70 kDa sTLR4 on the inhibitory activity of Stx2a on Raji cell translation. Preincubation: Stx2a (0.5 nM) was preincubated in the absence and in the presence of HuSAP (0.5 μM) or 70 kDa sTLR4 (0.5 μM) in 22.5 μL PBS for 10 min at 37 °C. After incubation, 10 μL aliquots were withdrawn and added to Raji cells (final volume 500 μL) to reach the final concentrations 10 pM Stx2a (IC_80_ on Raji cell translation), 10 nM HuSAP, and 10 nM 70 kDa sTLR4. Where indicated Stx2a (10 pM), HuSAP (10 nM), and 70 kDa sTLR4 (10 nM) were directly added to the assay. The data are expressed as percentage activity (mean ± SD, two-tailed paired *t*-test, *n* = 4), * *p* < 0.01. +: addition of the indicated compounds.

To study the effect of 70 kDa sTLR4 on the biological activity of Stx2a, we used Stx-sensitive human cells that express Gb3Cer, namely Raji cells. According to a previously described method [32], cells were treated for a short time (3 h) with Stx, and then cell translation was measured after the addition of a radioactive amino acid. The extent of protein synthesis inhibition with respect to control untreated cells was determined. The presence of 70 kDa sTLR4 (5 μg/mL) or of the complex 70 kDa sTLR4/MD2 (5 μg/mL) did not modify the IC_50_ of Stx2a on protein synthesis in Raji cells (Table 1), suggesting that a putative complex between Stx2a and cell-free soluble TLR4 is active in intoxicating target cells. Conversely, it is well known that Stx2a, but not Stx1a, acting on the same cellular model was greatly impaired by the presence of human serum containing HuSAP [32]. The effect of HuSAP on Stx2a is dramatic since even low concentration of purified HuSAP (10 nM) significantly impaired the cytotoxicity of Stx2a on Raji cells (Table 1). To verify if the binding of soluble TLR4 to Stx2a allows the toxin to escape from the capture by HuSAP, we pre-incubated Stx2a (0.5 nM) with a 1000 molar excess of both factors in different combinations and, after complexes formation, aliquots of the mixture were withdrawn and added to Raji cells to obtain a concentration of Stx2a (10 pM) approximately equal to its IC_80_, the inhibitor concentration that decreases translation by 80%. The strong protection induced by HuSAP was significantly alleviated by pre-incubating Stx2a with 70 kDa sTLR4 (Figure 2). Moreover, the simultaneous addition of HuSAP, 70 kDa sTLR4, and Stx2a to the assay allowed similar effects (Figure 2). Thus, the soluble form of TLR4 may impair HuSAP protection by creating physical hindrance for binding between HuSAP and Stx2a. It should be noted that if the opposite experiment was performed, i.e. pre-incubation of the toxin with HuSAP and then addition of 70 kDa sTLR4 to the assay, no action of the toxin was permitted. Hence, soluble TLR4 is not able to displace the toxin from the HuSAP/Stx2a complex. One can conclude that the simple release of soluble TLR4 in patients′ sera after monocyte/neutrophil challenge induced by LPS or Stx would have no effect once the binding of HuSAP with Stx2a had occurred. On the other hand, soluble TLR4 could interfere with HuSAP binding to freshly entered Stx2a in blood stream from the intestine. Although these experiments allowed us to conceptualize the reciprocal relationship between Stx2a and protective and/or inhibitory factors, they have been performed in conditions far from those observed in human sera. Thus, we calculated the IC_50_ of Stx2a on Raji cell translation in the presence of normal human serum. It was shown that simultaneous addition of Stx2a and high concentration of 70 kDa sTLR4 (10 nM) to Raji cells containing 10% human serum had no effect (Table 1) on IC_50_ compared with Raji cells without adding soluble TLR4. Conversely, the addition of Stx2a pre-incubated with 70 kDa sTLR4 significantly decreased the IC_50_ (Table 1). Therefore, a substantial increase of Stx2a toxicity would be achieved if TLR4/Stx2a complex is formed before Stx2a interacts with HuSAP.

## 3. Discussion

In this paper the effects of the extracellular soluble domain of TLR4 on the biological activity of Stx and Stx interaction with blood components (neutrophils, HuSAP) involved in the pathogenesis of HUS have been investigated. The soluble form of TLR4 was found to impair neutrophil/Stx interactions. However, high concentrations of 70 kDa sTLR4 were required to achieve this negative modulating function: 600 nM (~40 μg/mL) when the receptors on neutrophils are 100% saturated (Figure 1) or 60 nM (~4 μg/mL) when the receptors are 50% saturated (data not shown). These high concentrations of sTLR4 appear well beyond the capability of circulating monocytes or neutrophils because the maximal concentrations of the top mediators induced by Stx in these cells usually range from ~1 ng/mL (CXCL8, neutrophils) to ~30 ng/mL (CXCL8, monocytes) [5]. Moreover, 60 nM 70 kDa sTLR4 corresponds to 3.6 × 10^13^ molecules per milliliter, while the average numbers of neutrophils and monocytes in a two-year-old child’s blood are about 3.5 × 10^6^ and 5 × 10^5^ per milliliter [33], respectively, suggesting each cell has to release 1–7 × 10^7^ sTLR4 molecules to reach the needed level, which is highly unlikely. Based on preliminary ELISA on sera from patients with STEC-induced HUS the concentration of the soluble form of TLR4 was nanogram per milliliter level (data not shown). Thus, the hypothesis that the soluble TLR4 would inhibit the binding of freshly entered Stx in blood from the gut to neutrophils seems unlikely during the natural course of STEC-infections. 

Our results demonstrated that cell-free TLR4 protected Stx2a from capturing by HuSAP in blood that inhibits Stx2a interaction with Gb3Cer on target cells. Once a Stx2a/HuSAP complex is formed, soluble TLR4 is no longer able to displace HuSAP. Conversely, formation of Stx2a/70 kDa sTLR4 complex allows the toxin to bind Gb3Cer. According to calculation of the reciprocal interactions between these factors based on the physiological concentrations of HuSAP and on experiments with human serum as source of HuSAP, it is also unlikely that soluble TLR4 would be able to prevent the formation of the Stx2a/HuSAP complex in patients’ blood.

A possible explanation for Stx2a forming complex with sTLR4 before interacting with HuSAP is that certain amounts of soluble TLR4 could be produced during the intestinal phase of STEC-infections by LPS- or Stx2a-stimulated macrophages resident in the lamina propria of the gut. In this case, the toxin would translocate through the intestinal barrier as Stx2a/sTLR4 complex. 

Another attractive hypothesis is that detachment of Stx2a already bound with TLR4 in circulating cells would allow Stx2a to escape from HuSAP negative modulating effect. This could happen (1) after proteolytic processing of the membrane TLR4 originating macromolecular complexes Stx2a/sTLR4 or (2) considering Stx2a as a membrane component bound to TLR4 present on microvesicles released by activated circulating cells after toxin challenge. It is worth noting that one of the most recent hypotheses on the pathogenesis of STEC-induced HUS proposed that Stx-containing microvesicles released by circulating cells may be the main trigger. In this model, circulating cells represent a reservoir of toxins available for the follow-up intoxication of the renal target cells after their release. At a molecular level, the binding of the extracellular domain of TLR4 exposed on microvesicles to Stx2a could protect toxin from binding by HuSAP, hence favoring its toxicity for target endothelial cells. As shown in Figure 2 and Table 1, the toxicity of Stx2a rescued by soluble TLR4 is only partial, however, over a sufficiently long period of time, this toxicity could accumulate to a level high enough to cause diseases. The role of blood cell-derived Stx-containing microvesicles and the relevance of the soluble form of TLR4 in HUS needs to be further validated in studies on patients during the natural course of STEC-infections.

## 4. Materials and Methods

### 4.1. Materials and Toxins

Stx1a produced by *E. coli* C600 (H19J) and Stx2a produced by *E. coli* C600 (933W) were purified by receptor analog affinity chromatography, on globotriose-Fractogel (IsoSep, Lund, Sweden) [34] and on (Gala1-4Galb-O-spacer)-BSASepharose 4B (Glycorex, Lund, Sweden) [35], respectively, followed in both cases by a passage through ActiClean Etox columns (Sterogene Bioseparations, Carlsbad, CA, USA) to remove trace endotoxin contaminant. HuSAP was purchased from Sigma-Aldrich (St. Louis, MO, USA); 70 kDa sTLR4 (human recombinant extracellular domain of TLR4), 70 kDa sTLR4/MD2 complex, and recombinant human TLR2 were obtained by R&D Systems (Minneapolis, MN, USA) and resuspended as described in the manufacturer′s instructions.

### 4.2. Experimental Binding of Shiga Toxins to Human Neutrophils

Highly pure neutrophils (99.7%) were isolated under endotoxin-low conditions from buffy coats of healthy donors after centrifugation over Ficoll-Paque, followed by dextran sedimentation, hypotonic lysis of contaminating erythrocytes, and removing of any eventual contaminating cells by using the EasySep human neutrophil enrichment kit (Stemcell Technologies, Vancouver, BC, Canada) [11,36]. Healthy donors gave their informed consent for the use of their blood samples in the experiments described in this paper. For binding experiments with purified Stx1a and Stx2a, Eppendorf tubes were precoated with PBS containing 1% endotoxin-low (≤1 endotoxin unit/mg) BSA to avoid nonspecific loss of the toxins. Neutrophils (5 × 10^5^) were incubated for 90 min at 37 °C with 60 nM Stx in 250 μL of the same buffer (PBS-BSA). After incubation, the cells were spun down at 200× *g* for 5 min and washed three times with 100 μL incubation buffer at 37 °C. Stx bound to neutrophils were detected by flow cytometry as describe in the paragraph below.

### 4.3. Detection of Shiga Toxins Bound to Neutrophils

Stx bound to neutrophils were detected by flow cytometry, as reported in a specific methodological article in which all technical details were carefully described [31]. The assay was validated by comparing control subjects and HUS patients in a blinded manner [31] and by challenging Stx-positive neutrophils with a negative control antibody [37]. Briefly, untreated or Stx-treated neutrophils were incubated with mouse monoclonal antibodies (IgG) against Stx1a (Stx1-13C4, Toxin Technology, Sarasota, FL, USA) or Stx2a (Stx2-BB12, Toxin Technology, Sarasota, FL, USA) in the presence of human serum to saturate Fc receptors on leukocytes. After incubation with FITC-goat anti-mouse IgG, flow cytometric analysis was used to reveal the neutrophil-bound fluorescence, allowing a highly sensitive detection of both Stx. Cells were visualized by a cytogram that combined forward scatter versus 90° side scatter, and fluorescence was analyzed by a cytogram that combined 90° side scatter and fluorescence and by a single-fluorescence histogram. Neutrophils were checked by staining with mAb to Ags associated to granulocytes (FITC-CD16 and FITC-CD65, Beckman Coulter, Miami, FL, USA). The mean channel value of fluorescence (MCV) was chosen as an objective parameter to measure the extent of binding of Stx to neutrophils.

### 4.4. Detection of the Functional Activity of Shiga Toxins in Whole Cells

The rapid intoxication kinetic of human Gb3Cer-expressing Raji cells and their high sensitivity to Stx were exploited for the detection of the toxic activity of Stx2a by measuring the inhibition of translation [32]. Protein synthesis was measured after 3 h incubation with different concentrations of Stx2a as the rate of incorporation of [^3^H] leucine into proteins during 60 min incubation in complete medium as described previously [32] in the absence or in the presence of pooled human serum from three healthy donors as source of HuSAP.

### 4.5. Statistical Analysis

Statistical analysis was performed with GraphPad Prism 5 software (GraphPad Software, La Jolla, CA, USA). Continuous variables were described through means and SD. Differences in continuous variables were tested with a *t*-test after controlling the normality of their distribution. Correlation between variables was assessed using the Pearson correlation coefficient. A *p* value < 0.05 was considered statistically significant.

## Figures and Tables

**Table 1:** Effects of the soluble extracellular domain of TLR4 or of HuSAP on the inhibition of translation induced by Stx2a in Raji cells.

Additions	IC_50_^a^ Stx2a Fold Increase	IC_50_ Determination (r)	Statistical Significance ^b^
None	1	−0.991	-
5 µg/mL sTLR4 (70 nM)	1	−0.999	n.s.
5 µg/mL sTLR4/MD2 (55 nM)	1	−0.999	n.s.
0.25 µg/mL HuSAP (1 nM)	1.6	−0.977	n.s.
2.50 µg/mL HuSAP (10 nM)	52.2	−0.999	*p* < 0.0001
10% human serum	20.7	−0.992	*p* < 0.0001
10% human serum + sTLR4 (10 nM)	21.0	−0.967	*p* < 0.0005
10% human serum + (Stx2a pre-incubated with sTLR4)	14.7 *	−0.999	*p* > 0.0001

^a^ IC_50_ were calculated by the linear regression between fractional activity and the log of Stx2a concentrations. ^b^ The statistical significance of the differences between the intercepts of the straight lines obtained with the indicated additions with respect to Stx2a alone (none) are reported (n.s., not significant). * Significant difference between the intercepts of the straight lines obtained in the presence of 10% human serum with Stx2a and with Stx2a/ 70 kDa sTLR4 complex (*p* < 0.05).
